# Autophagy changes in lung tissues of mice at 30 days after carbon black‐metal ion co‐exposure

**DOI:** 10.1111/cpr.12813

**Published:** 2020-06-09

**Authors:** Wei He, Hongzhen Peng, Jifei Ma, Qisheng Wang, Aiguo Li, Jichao Zhang, Huating Kong, Qingnuan Li, Yanhong Sun, Ying Zhu

**Affiliations:** ^1^ Division of Physical Biology and Bioimaging Center Shanghai Synchrotron Radiation Facility Shanghai Institute of Applied Physics Chinese Academy of Sciences Shanghai China; ^2^ University of Chinese Academy of Sciences Beijing China; ^3^ Zhangjiang Laboratory Shanghai Advanced Research Institute Chinese Academy of Sciences Shanghai China; ^4^ Key Laboratory of Interfacial Physics and Technology Shanghai Institute of Applied Physics Chinese Academy of Sciences Shanghai China; ^5^ Shanghai Synchrotron Radiation Facility Shanghai Institute of Applied Physics Chinese Academy of Sciences Shanghai China

**Keywords:** autophagy, carbon blacks (CBs), fine particulate matter (PM2.5), lung, metal

## Abstract

**Objectives:**

Accumulating studies have investigated the PM2.5‐induced pulmonary toxicity, while gaps still remain in understanding its toxic mechanism. Due to its high specific surface area and adsorption capacity similar to nanoparticles, PM2.5 acts as a significant carrier of metals in air and then leads to altered toxic effects. In this study, we aimed to use CBs and Ni as model materials to investigate the autophagy changes and pulmonary toxic effects at 30 days following intratracheal instillation of CBs‐Ni mixture.

**Materials and methods:**

Groups of mice were instilled with 100 µL normal saline (NS), 20 µg CBs, and 4 µg Ni or CBs‐Ni mixture, respectively. At 7 and 30 days post‐instillation, all the mice were weighed and then sacrificed. The evaluation system was composed of the following: (a) autophagy and lysosomal function assessment, (b) trace element biodistribution observation in lungs, (c) pulmonary lavage biomedical analysis, (d) lung histopathological evaluation, (e) coefficient analysis of major organs and (f) CBs‐Ni interaction and cell proliferation assessment.

**Results:**

We found that after CBs‐Ni co‐exposure, no obvious autophagy and lysosomal dysfunction or pulmonary toxicity was detected, along with complete clearance of Ni from lung tissues as well as recovery of biochemical indexes to normal range.

**Conclusions:**

We conclude that the damaged autophagy and lysosomal function, as well as physiological function, was repaired at 30 days after exposure of CBs‐Ni. Our findings provide a new idea for scientific assessment of the impact of fine particles on environment and human health, and useful information for the comprehensive treatment of air pollution.

## 1 INTRODUCTION

Exposure to PM2.5 has been found to be associated with adverse health effects, including cardiovascular disease, respiratory irritation and pulmonary dysfunction,^1^, ^2^, ^3^, ^4^ yet gaps still remain in understanding the comprehensive mechanisms of PM2.5‐induced pulmonary toxicity. Autophagy, a dynamic subcellular process regulating cellular homeostasis and adaptation to adverse conditions, has been considered as a molecular mechanism mediating the PM2.5‐induced pulmonary toxicity.^5^, ^6^ Nevertheless, current understanding of the toxic mechanism of PM2.5 mediated by autophagy is still limited. Further study of autophagy changes as well as long‐term systematic biocompatibility assessment following exposure of PM2.5 is essential.

To better understand the toxicity and mechanisms of PM2.5, nanoparticles with sub‐micro nanometres and similar biological activities (including toxic effects, cellular uptake pathways and toxicity mechanisms) were considered as predictable models.^7^, ^8^, ^9^, ^10^ Due to its high specific surface area and adsorption capacity similar to nanoparticles, PM2.5 acts as a significant carrier of multipollutant in air, and then leads to altered toxic effects, including synergistic airway inflammation and pulmonary impairment.^11^, ^12^, ^13^, ^14^ The interaction of PM2.5 and the anthropogenic metals in air is particularly relevant to human health.^15^, ^16^, ^17^ Thus, the interaction between PM2.5 and metals should also be considered in study of the toxic mechanism of PM2.5 mediated by autophagy. Recently, we used CBs and metal ions as mimicking model materials to investigate the synergistic pulmonary effects and its mechanism.^10^ We found co‐exposure to CBs and metals caused a synergistic pulmonary toxic effect attributed to autophagy and lysosomal dysfunction at 1 and 7 days post‐instillation in mice. Yet, an improved understanding of the long‐term changes in autophagy and toxicity after co‐exposure to CBs and metals is essential. Autophagy and lysosomal function assessment, as well as toxicological investigations, will be needed for evaluating the comprehensive lung function following exposure of CBs‐metal ions.

In the present study, we investigated the autophagy changes and pulmonary toxic effects in mice at 30 days following intratracheal instillation of CBs‐metal ion. We demonstrated that 30 days after an exposure to CBs‐metal ion has ended, no obvious autophagy and lysosomal dysfunction or inflammatory response was detected, along with complete clearance of metal ion as well as recovery of biochemical indexes to normal range. Therefore, we indicate that the damaged autophagy and lysosomal function, as well as physiological function in 7 days post‐exposure, could be completely repaired at 30 days following an exposure of CBs‐metal ion.

## 2 METHODS AND MATERIALS

### 2.1 Materials

**Figure 1:**
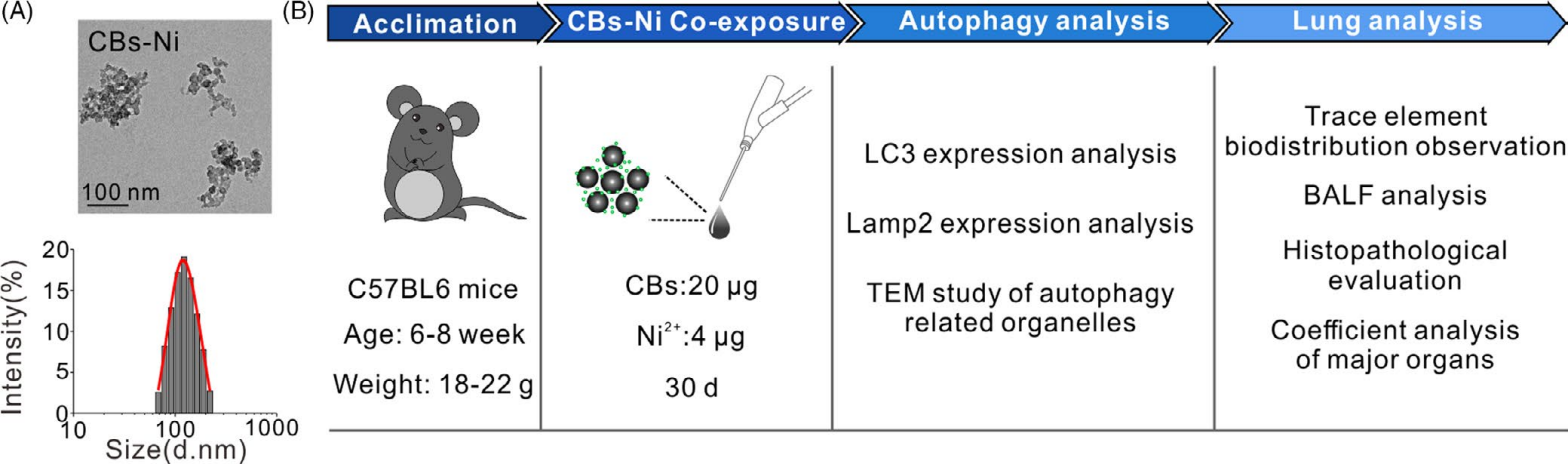
Materials and experimental design. A, TEM image and DLS measurement statistics of CBs‐Ni. B, Experimental design. C57BL6 mice were acclimated for 5 d before initiating the experimental protocol. The mice were intratracheally instilled with NS (100 µL), CBs (20 µg), Ni (4 µg) or CBs‐Ni mixture. At 30 d after instillation, the autophagy function and pulmonary toxicity in lung were evaluated. The evaluation system was composed of the following (a): autophagy and lysosomal function assessment, (b) trace element biodistribution observation in lungs, (c) pulmonary lavage biomedical analysis, (d) lung histopathological evaluation, (e) coefficient analysis of major organs and (f) CBs‐Ni interaction and cell proliferation assessment

CBs with individual size of 14 nm were purchased from Degussa. CBs were dispersed in distilled water and then sonicated for 20 minutes to obtain well‐dispersed CB stock solution. Resultant suspension was stable for extended periods of time. CBs and CBs‐Ni mixture were well characterized by TEM and dynamic light scattering (DLS) (Figure 1A, Table S1, Figure S1). The details for characterization have been described in our previous work.^10^

NiCl_2_.6H_2_O was obtained from Sigma‐Aldrich. A stock solution of NiCl_2_.6H_2_O was prepared with distilled water and was used to make serial dilutions. All other chemicals used were of analytical grade.

### 2.2 Animals and treatment

C57BL6 mice (male, 18‐22 g) were purchased from Shanghai SLAC Laboratory Animal Co. Ltd., and kept in conventional conditions (18‐22°C, 50%‐70% relative humidity, 12‐hour light‐dark cycle). The animals were acclimated for 5 days before initiating the experimental protocol. All animal experiments were conducted in accordance with the Institute's Guide for the Care and Use of Laboratory Animals and were approved by the ethical committee of Shanghai University of Traditional Chinese Medicine (Approval No. ACSHU‐2014‐200, approved on 16 July 2014).

A total of 40 C57BL6 mice were randomly divided into 5 groups of 8 each NS (30 days), CBs (30 days), Ni (30 days), CBs‐Ni mixture (30 days) and CBs‐Ni mixture (7 days). Groups of mice were instilled with 100 µL normal saline (NS), 20 µg CBs, 4 µg Ni or CBs‐Ni mixture (20 µg CBs mixed with 4 µg Ni), respectively. All samples were suspended in NS containing 0.05% Tween 80. At 7 and 30 days post‐instillation, animals in all groups were weighed and then sacrificed by eyeball‐extracted methods. The evaluation system was composed of the following: (a) autophagy and lysosomal function assessment, (b) trace element biodistribution observation in lungs, (c) pulmonary lavage biomedical analysis, (d) lung histopathological evaluation, (e) coefficient analysis of major organs and (f) CBs‐Ni interaction and cell proliferation assessment (Figure 1B).

### 2.3 Intratracheal instillation

Intratracheal instillation was performed using a rapid, non‐surgical and efficient method developed in our previous work. Briefly, the experimental mice were fully anaesthetized and hung on the bracket by hooking their incisors with silk. Particulate suspensions were pipetted into mouse lung directly via glottidis rimae under the irradiation of cold light source.

### 2.4 Synchrotron‐based X‐ray fluorescence microscopy

Synchrotron‐based X‐ray fluorescence (XRF) microscopy was used to investigate in vivo fate of CBs‐Ni at 30 days after intratracheal instillation. Optimal cutting temperature compound (oct)‐embedded lung sections (30 µm thick) were placed on Mylar X‐ray films. The micro‐X‐ray fluorescence (μXRF) microscopy was performed at the beamline BL15U1 of SSRF. Incident X‐ray energy of 12 keV, obtained with a Si (111) monochromator, was chosen in order to excite the K‐lines of X‐ray fluorescence of elements Ni, Fe, Cu, Zn and Ca. A light microscope was coupled to a computer for sample viewing, and the sample platform was moved by a motorized X‐ray mapping stage. A Kirkpatrick‐Baez mirror system focused the X‐ray beam to a spot size of 150 × 150 μm on the specimen, which was raster‐scanned. XRF from the specimen was captured with an energy‐dispersive silicon drift detector (Vortex). From the analysis of the X‐ray fluorescence spectrum for each pixel, a spatial image can be obtained for each element separately. Such an image represents a two‐dimensional projection of the volumetric distribution of the elements. The vertical and horizontal pixel size was 150 μm each. Data collection time for each pixel was 2 secconds, and fitting of the fluorescence data has been performed in batch processing using the pymca 4.0.9 software.

### 2.5 Cell line and treatment

RAW264.7 macrophage‐like cells were cultured (37°C, 5% CO_2_) in the Dulbecco's modified Eagle's medium (DMEM) with 10% foetal bovine serum (FBS) and antibiotics (100 units/mL penicillin and 100 μg/mL streptomycin). RAW264.7 cells were seeded in 24‐well plates at a density of 3 × 10^4^ cells/well and incubated 8‐12 hours to allow for adherence. Following phosphate‐buffered saline (PBS) washing, the cells were treated with CBs‐Ni mixture for 1‐8 days. The cell culture medium was replaced with fresh one every 2 days. Ni solution was mixed thoroughly with the aqueous CB solution for 2 hours at 37°C prior to experiments. Cells incubated with the complete culture medium were used as control.

### 2.6 Western blotting analysis

After treatment, lung tissues excised from mice after treatment were minced and homogenized in protein lysate buffer. Debris was removed by centrifugation, and the protein samples were analysed by 10% or 15% SDS‐PAGE (as appropriate) and blotted to PVDF membranes. The blots were blocked for 30 minutes using 6% nonfat milk in PBST (PBS containing 0.1% Tween 20) buffer and then incubated overnight at 4°C with the primary antibodies as required: anti‐LC3B (1:1000 dilution, Novus), Lamp2 (1:1000 dilution, Abcam) or GAPDH (1:1000 dilution, Abcam). After extensive washing, the blots were probed with a goat anti‐rabbit horseradish peroxidase‐conjugated antibody (1:10 000 dilution, KPL) for 1 hour. The blots were then developed by incubation with chemiluminescence (ECL) plus and exposed to X‐ray film. The densities of all bands were quantified with a computer densitometer (AlphaImager^™^ 2200 System Alpha Innotech Corporation, GBBOX‐Chemi‐XL1.4). The expression of GAPDH was used as the protein loading control. Original uncropped scans of Western blots included in main figures are shown in Figure S7.

### 2.7 Transmission electron microscopy

After treatment, lung tissue bits excised from mice were immediately fixed in 2.5% glutaraldehyde in 0.1 mol/L phosphate buffer (PB) and stored at 4°C until embedding. Ultrathin sections of the embedded cells and tumour tissues were examined by transmission electron microscope (TEM, JEOL‐1230; JEOL).

### 2.8 Inductively coupled plasma‐mass spectrometry (ICP‐MS)

For adsorption kinetic experiments, Ni solution (500 µg/mL) was mixed thoroughly with the CB solution (1 mg/mL) at a v/v ratio of 1:1 for 10 minutes, 30 minutes, 1 hour, 2 hours, 1 day, 2 days and 30 days at 37°C. After centrifugation at 21,000 *g* for 30 minutes, the concentration of Ni in the supernatant was determined by ICP‐MS (x series 2, Thermo Scientific). The amounts of Ni adsorbed on the CBs were calculated by subtraction.

For in vivo experiments, after treatment, tissues including heart, liver, spleen, lung and kidney were digested by HNO_3_ and H_2_O_2_ mixture (v/v ratio is 7:1) at 130°C until the mixed solutions became colourless and clear. The Ni concentration in all samples was analysed by ICP‐MS.

### 2.9 Pulmonary lavage and biomedical analysis

After treatment, the mice lungs were lavaged twice with 2 mL phosphate‐buffered saline passed through a cannula anchored in the trachea. Bronchoalveolar lavage fluid (BALF) was centrifuged at 450 *g* at 4°C for 10 minutes to pellet cells. Lactate dehydrogenase (LDH), alkaline phosphatase (ALP) and acid phosphatase (ACP) in supernatant were assayed with the detection kits (Nanjing Jiancheng Bioengineering Institute) according to the instructions. The results were expressed as IU/L.

### 2.10 Histopathological analysis of lung tissues

Paraffin‐embedded lung sections of mice were stained with haematoxylin and eosin and examined by optical microscopy. The pathologist performing the visual analysis was blind to the grouping of mice.

### 2.11 Coefficient analysis of major organs

After weighing the body and tissues, the coefficients of liver, spleen, lung and kidney to body weight were calculated as the ratio of tissues (wet weight, mg) to body weight (g).

### 2.12 Cell proliferation assay

Cell proliferation of RAW264.7 cells was evaluated by determining growth curves. After treatment, cells were washed by PBS, trypsinized, stained with trypan blue, and the cell number at each time point was determined by an automated cell counter (Countstar). All data were based on three independent measurements.

### 2.13 Observation for cellular uptake of CBs‐Ni by optical microscopy

After treatment, the cells were washed with PBS and all samples were observed under an Olympus CKX41 inverted optical microscope (Olympus).

### 2.14 Statistical analysis

All results are expressed as the mean ± standard deviation from triplicate experiments performed in a parallel manner unless otherwise indicated. Statistical significance of the data was determined by *t* tests or one‐way analysis of variance (ANOVA) using spss 11.5. *equals *P* < .05; **equals *P* < .01.

## 3 RESULTS

### 3.1 Autophagy and lysosomal function assessment

The autophagy‐lysosomal pathway plays a critical role in maintaining intercellular homeostasis under PM2.5 exposure, including encapsulation of fine particles, damaged proteins and organelles by autophagic vesicles, followed by lysosomal degradation and recycling.^10^, ^18^, ^19^ Autophagy and lysosomal dysfunction has been considered as an important molecular mechanism of PM2.5‐mediated pulmonary toxicity. In our previous study, we investigated the interaction between CBs and Ni, Cu, Cd and Cr We found that the adsorption amounts of CBs for different ions were different, and the adsorption for Ni was the strongest among those metal ions, which are responsible for its most significant synergetic effect. Therefore, Ni was chosen as the representative of anthropogenic metals. We found that CBs‐Ni co‐exposure caused autophagy and lysosomal dysfunction in mice lungs at 1 and 7 days post‐instillation.^10^ However, the change in autophagy and lysosomal function in lung tissues after a longer period following exposure of CBs‐metal ions has not been explored.

**Figure 2:**
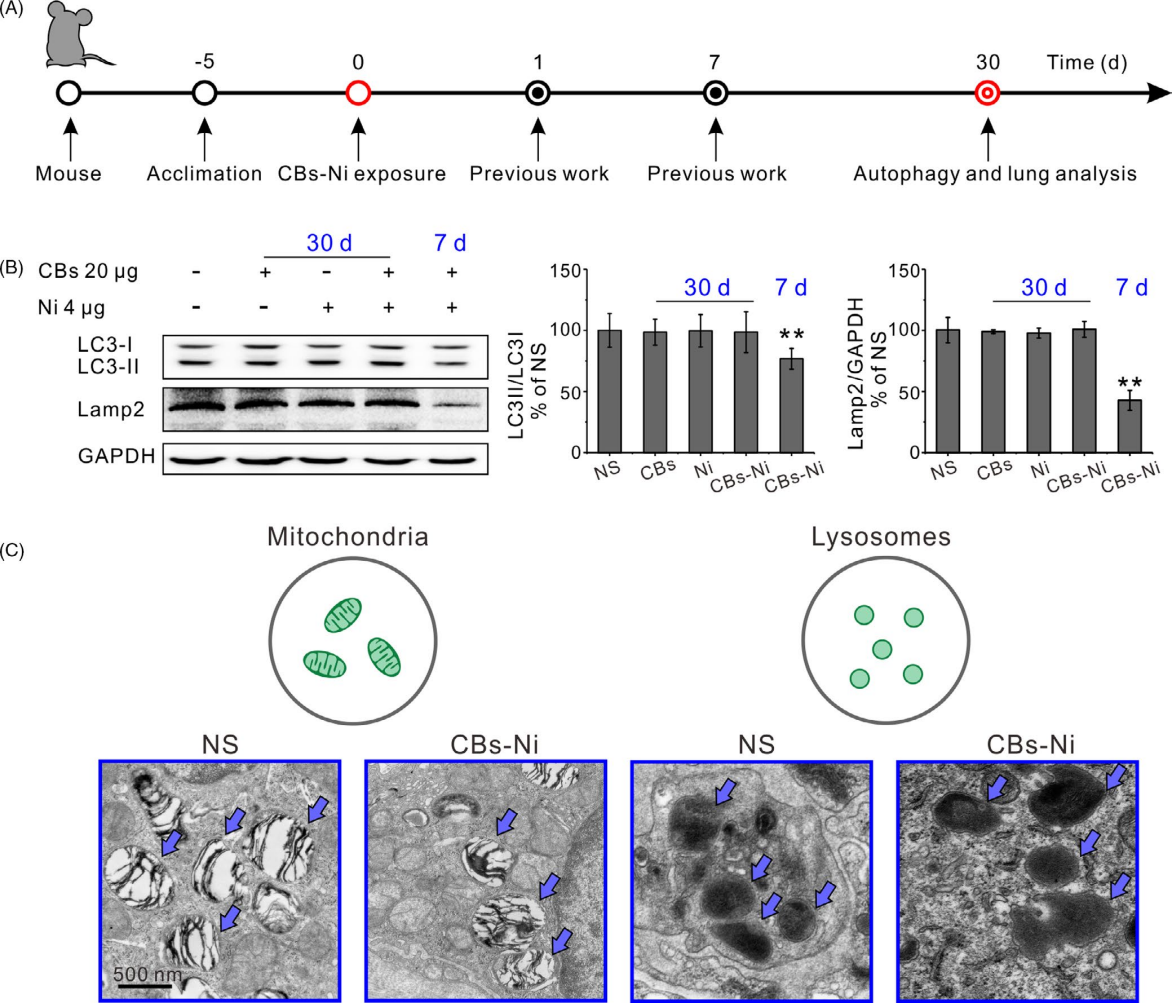
Repaired autophagy and lysosomal function in lung tissues. C57BL6 mice were intratracheally instilled with NS (100 µL), CBs (20 μg), Ni (4 μg) or CBs‐Ni mixture. A, Experimental design. B, Representative immunoblots of LC3‐Ⅰ, LC3‐Ⅱ and Lamp2, and their quantification (n = 3) in lung tissues of mice at 30 d post‐instillation. ^**^ *P* < .01, significantly different from NS. C, TEM images of lung tissue sections of mice at 30 d post‐instillation. Mitochondria and lysosomes are indicated with blue arrows

In the current study, we assessed the change in autophagy and lysosomal dysfunction caused by acute exposure of CBs‐metal ions in lung tissues. The CBs‐Ni (cluster size of 118 nm) used in this study was characterized by TEM and DLS (Figure 1A). C57BL6 mice were intratracheally instilled with NS (100 µL), CBs (20 µg), Ni (4 µg) or CBs‐Ni mixture by using a rapid, non‐surgical and efficient method developed in our previous work (Figure S2). The autophagosomal marker LC3‐II/LC3‐I ratio could reflect the autophagic activity,^20^ and the Lamp2 is a major lysosomal membrane protein involved in lysosomal stability and autophagy.^21^, ^22^ Here, we analysed the level of LC3‐II/LC3‐I ratio and Lamp2, to monitor the autophagy and lysosomal function (Figure 2A). Western blot analysis indicated that at 30 days post‐instillation, CBs‐Ni co‐instillation as well as individual CBs and Ni did not change the level of LC3‐II/LC3‐I ratio or Lamp2 significantly. As a contrast, at 7 days post‐instillation, CBs‐Ni co‐instillation induced indeed decrease in expression of LC3‐II and notably degradation of Lamp2, demonstrating a severe autophagy and lysosomal dysfunction (Figure 2B). TEM images revealed that co‐instillation did not change the mitochondria morphology and lysosomal membrane integrity (Figure 2C). In contrast, we found obvious disruption of the mitochondria morphology inside lung cells at 7 days post‐exposure of CBs‐Ni mixture (Figure S3). All these data indicate that the damaged autophagy and lysosomal function at 7 days post‐instillation has been repaired completely at 30 days post‐instillation.

### 3.2 Trace element biodistribution observation in lungs

**Figure 3:**
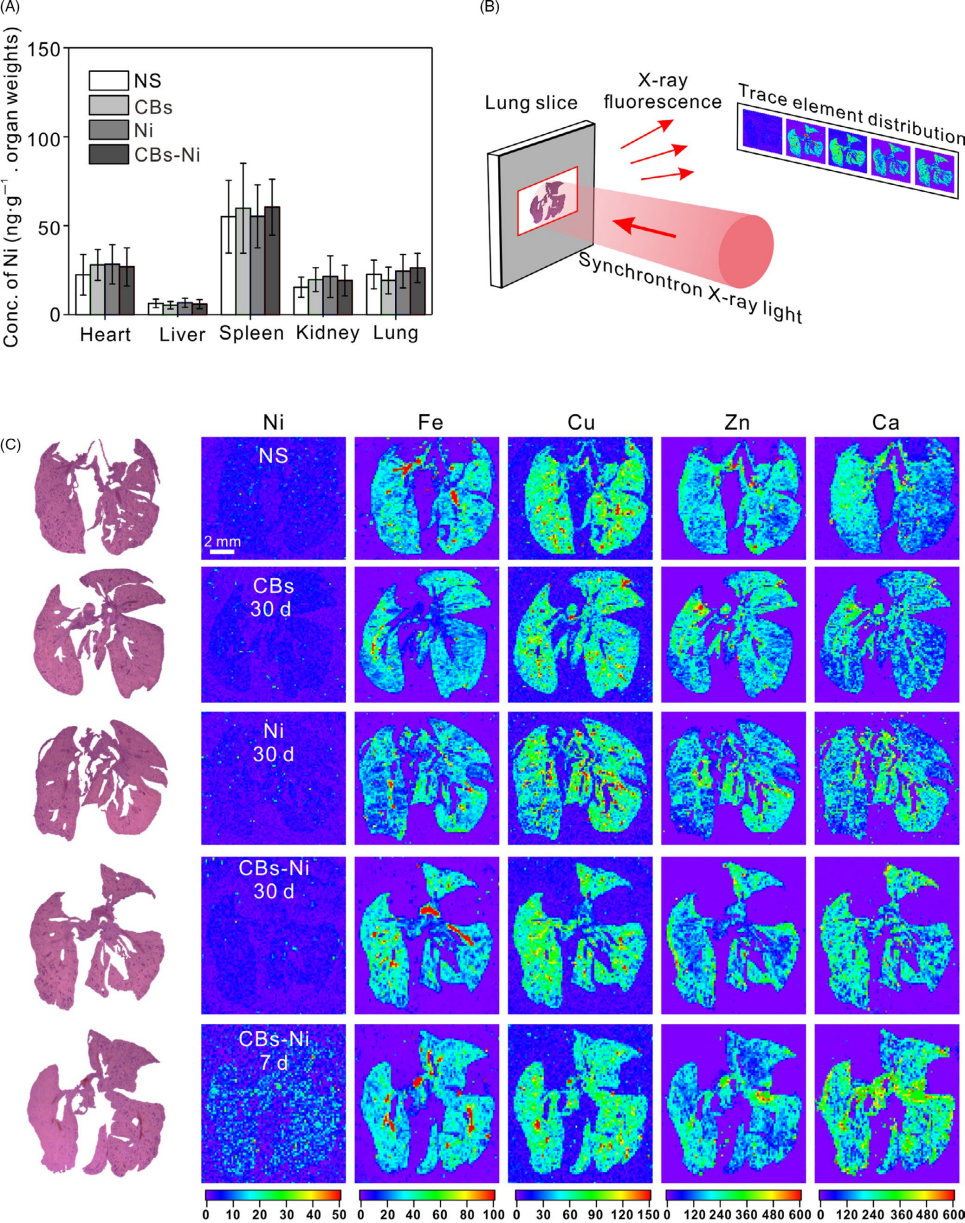
Trace element biodistribution observation in lungs. C57BL6 mice were intratracheally instilled with NS (100 µL), CBs (20 μg), Ni (4 μg) or CBs‐Ni mixture. A, Ni concentration in the major organs of mice at 30 d post‐instillation determined by ICP‐MS. Data are represented as means ± SD. B, Schematic showing of trace element biodistribution observation by XRF. C, XRF images of Ni, Fe, Cu, Zn and Ca distribution in lung tissues of mice at 30 d post‐instillation. Each XRF image (right) is paired with its respective histological image (left)

To further investigate the clearance of metal ion in mice 30 days post‐exposure, we measured the concentration of Ni element in heart, liver, spleen, kidney and lung by ICP‐MS analysis. We found that, at 30 days post‐instillation, there was no significant difference in Ni concentration of major organs among all groups (Figure 3A). Thus, we consider that excessive Ni could be cleared from the body at 30 days post‐instillation. Apart from Ni, the in vivo distribution and concentration of several trace elements (including Fe, Cu, Zn and Ca) which are indispensable for maintaining life activities are important biomarkers of body's physiological functions.^23^, ^24^, ^25^ Over the past few years, we have performed a series of studies on elements tracking in vitro or in vivo by using synchrotron‐based X‐ray fluorescence (XRF) microscopy.^26^, ^27^, ^28^, ^29^ Here, we employed XRF microscopy to observe the biodistribution of Ni, Fe, Cu, Zn and Ca elements in lungs (Figure 3B). XRF images showed that at 30 days post‐instillation, there was no obvious change in the concentration and distribution of Ni, Fe, Cu, Zn and Ca elements in lungs compared with that in normal lungs. As a contrast, at 7 days post‐instillation, CBs‐Ni co‐instillation increased the Ni and Ca levels, demonstrating an acute lung injury (Figure 3C). Their results suggest that there was no abnormality in the biodistribution of these essential elements in lungs at 30 days post‐instillation.

### 3.3 Examination of pulmonary toxicity of CBs‐metal ion co‐exposure

**Figure 4:**
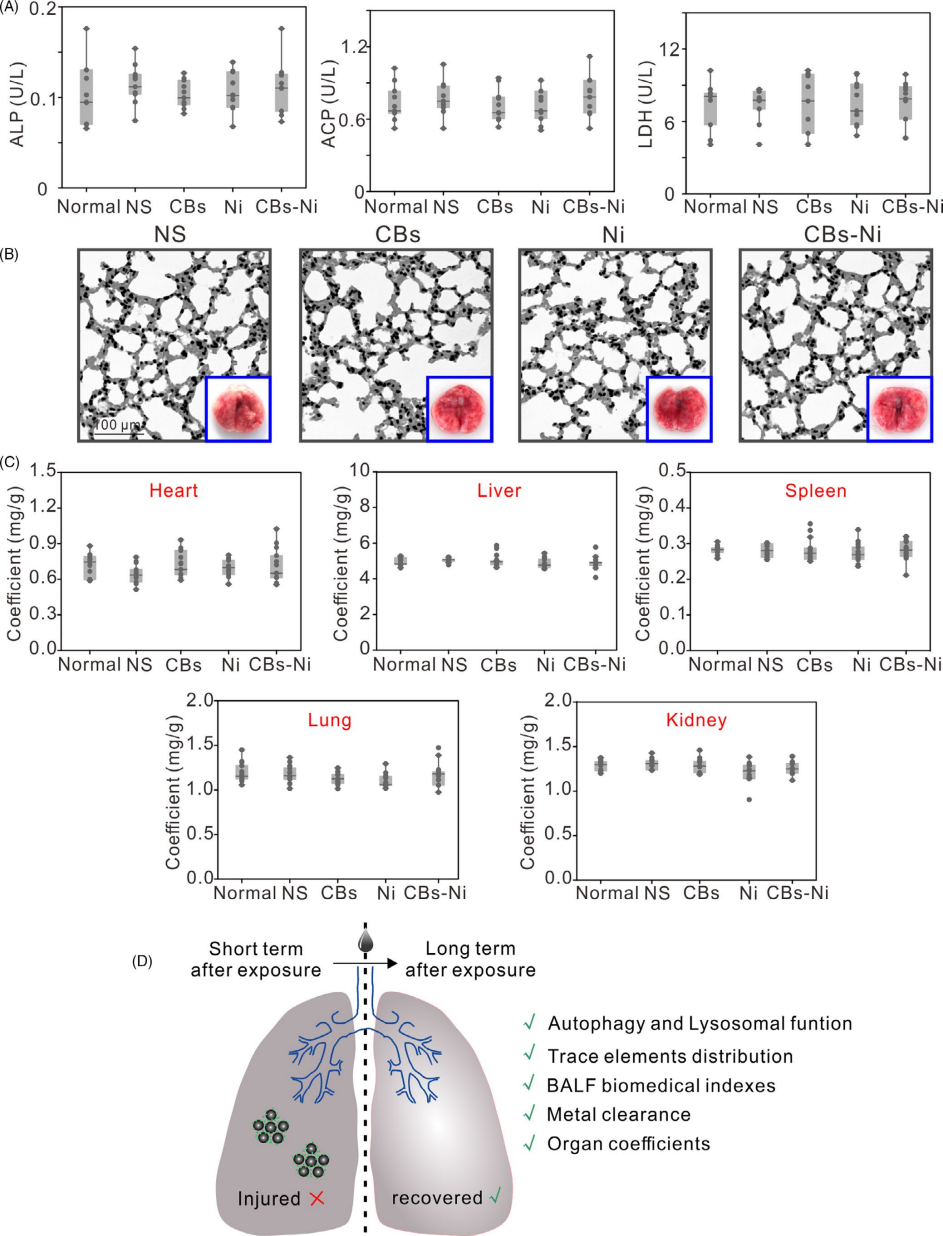
Pulmonary toxicity of CBs‐Ni co‐exposure. C57BL6 mice were intratracheally instilled with NS (100 µL), CBs (20 μg), Ni (4 μg) or CBs‐Ni mixture. A, Biochemical parameters in BALF (n = 8) in mice at 30 d post‐instillation. Data are represented as means ± SD. B, Haematoxylin and eosin (H&E) histopathological sections of lung tissues were analysed at 30 d post‐instillation (n = 3). Insets are morphological observation of the lungs from mice after corresponding treatment. C, Coefficient (n = 8) of major organs of mice at 30 d post‐instillation. Data are represented as means ± SD. D, Schematic overview of long‐term lung recovery following exposure of CB‐Ni

**Figure 5:**
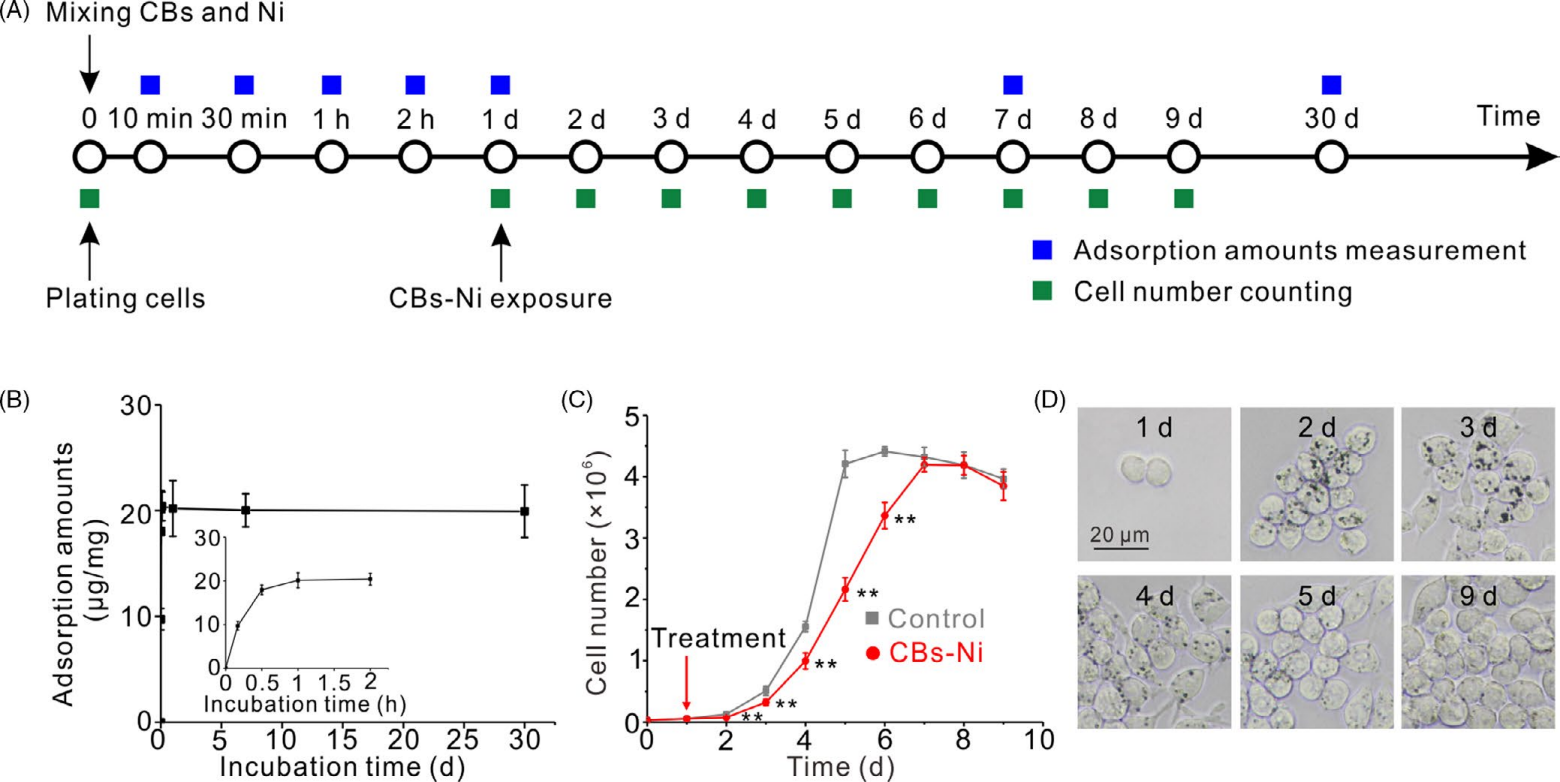
Long‐term cellular effects of CBs‐Ni interaction. A, Experimental design. B, Adsorption kinetics of Ni on CBs determined by ICP‐MS. Data are represented as means ± SD. C, Growth curves of RAW264.7 cells after incubation with CBs‐Ni. Data are represented as means ± SD. ^**^ *P* < .01, significantly different from control. D, Optical microscope images of RAW264.7 cells after incubation with CBs‐Ni

We then assessed the pulmonary toxicity of CBs‐Ni co‐exposure. The doses of CBs (20 µg) and Ni (4 µg) have been shown to be minimal pulmonary toxicity to mice.^30^, ^31^ Biomedical indexes as markers for cell damage and pulmonary responses were assessed in the BALF at 30 days post‐instillation. The LDH, ALP and ACP activities in BALF indicate the lung cell membrane permeability, the number or activity of type II cells in the alveolar space and phagocytosis activity of polymorphonuclear leucocytes and macrophages, respectively.^32^, ^33^ The results show that there were no significant differences in BALF biomedical indexes, including LDH, ALP and ACP activity in NS‐, CBs‐, Ni‐ or CBs‐Ni mixture‐treated groups compared with that in normal groups (Figure 4A), suggesting no lung cell toxicity.^34^ To further evaluate the synergistic pulmonary toxicity of CBs‐Ni co‐exposure, we performed histological analysis of lungs from the mice 30 days post‐exposure. We found that mice in all groups showed minimal pathological changes including neutrophil infiltration or alveolar septa thickening in lungs (Figure 4B, Figures S4 and S5). In contrast, histological analysis of lungs from the mice at 7 days post‐CBs‐Ni co‐exposure showed obvious neutrophil infiltration or alveolar septa thickening in lungs (Figure S6). Additionally, no significant change in the morphology of lung tissues (Figure 4B) and coefficient of major organs (Figure 4C) further confirmed that CBs‐metal ion co‐exposure did not cause notable synergistic pulmonary toxicity. Combing with autophagy and lysosomal function assessment and trace element biodistribution observation in lungs, we conclude that the injured autophagy and lysosomal function, as well as physiological function at 7 days after CBs‐Ni co‐exposure, could be repaired completely, at 30 days post‐exposure (Figure 4D). Further, we investigated the CBs‐Ni interaction (Figure 5A). Adsorption kinetic results show that the adsorption amounts of Ni on CBs reached equilibrium after 2 hours, and kept stable for 30 days (Figure 5B). Therefore, we conclude that the CBs‐Ni interaction did not induce obvious pulmonary toxicity, at 30 days post‐instillation. Subsequently, we choose a phagocytic cell line (RAW264.7) as a representative cell model of alveolar macrophages, which is a main biological target in lung for inhaled PM,^35^, ^36^ to investigate the effect of CBs‐Ni on proliferation of RAW264.7 cells (Figure 5A). By cell counting assay, we found that after treatment, the growth rate of the CBs‐Ni‐treated RAW264.7 cells is significantly slower than that of untreated HeLa cells, indicating significant cytotoxicity in the short term post‐treatment. Notably, after 7 days treatment, the number of CBs‐Ni‐treated RAW264.7 cells had reached that of untreated RAW264.7 cells, suggesting the recovery of cell viability (Figure 5C). Additionally, we analysed the time‐dependent uptake of CBs‐Ni in RAW264.7 cells by optical microscopy. After 1 and 2 days of incubation, images show that there are dark aggregates inside cells, indicating obvious cell uptake of CBs‐Ni over time (Figure 5D). However, after 3 days incubation, we clearly observed a notable decrease in intracellular dark aggregates over time (Figure 5D), suggesting sustain exclusion of intracellular CBs‐Ni by exocytosis,^37^, ^38^ which may be an important reason for the long‐term cell viability recovery. These in vitro data are compatible with the in vivo evidences, demonstrating that the toxicity induced by CBs‐Ni could be repaired after a long period post‐exposure.

## 4 DISCUSSION

Nowadays, the potential for human exposure to PM2.5 is increasing, thus raises concerns about mitigating the adverse health effect of PM2.5.^39^, ^40^, ^41^, ^42^ As an important molecular mechanism mediating the PM2.5‐induced toxicity, autophagy has emerged as a promising target for the therapy of PM2.5‐induced pulmonary diseases.^43^, ^44^, ^45^ Nevertheless, current understanding of the autophagy changes following exposure of PM2.5 remains to be improved. Here, our work demonstrated that 30 days after exposure to CBs‐metal ion has ended, injured autophagy and lysosomal function, as well as physiological function at 7 days after CBs‐Ni co‐exposure, could be repaired completely. This phenomenon further illustrated that relying on the body's own clearing and immune system, the damaged autophagy‐lysosomal pathway caused by PM2.5 in the short term post‐exposure could be recovered, at the long term post‐exposure. Certainly, 30 days is a relatively long period for PM2.5 toxicity research, to better understand the autophagy and toxicity of PM2.5, comprehensive research at extended period post‐exposure should be further developed. Our finding provided two valuable insights into the therapy of PM2.5‐induced pulmonary diseases: (a) after an acute exposure of PM2.5, avoiding re‐exposure to PM2.5 is an effect means for protecting lung recovery, considering the injured lung function need to be recovered in a period and (b) it would be more effective to administer autophagy modulators in the early phase post‐exposure, due to the significantly induced autophagy dysfunction.

In conclusion, we used CBs and Ni as model materials to investigate the autophagy changes in lungs at 30 days following exposure of CBs‐Ni. Results showed that 30 days after exposure to CBs‐Ni has ended, no obvious autophagy and lysosomal dysfunction or inflammatory response was detected, along with complete clearance of Ni. We indicate that damaged autophagy and lysosomal function, as well as physiological function at 7 days post‐instillation, could be repaired completely, at 30 days post‐instillation. Finally, we performed an in vitro research on the long‐term cellular effects of CBs‐Ni interaction. These in vitro data are compatible with the in vivo evidences, demonstrating that the toxicity induced by CBs‐Ni could be repaired after a long period post‐exposure. To some extent, our findings would alleviate people's fear of PM2.5‐related toxic effects. More importantly, our findings provide new insights into understanding the lung recovery and its molecular mechanisms following exposure of PM2.5, which will contribute to the guidance of the scientific therapy of PM2.5‐induced pulmonary diseases and comprehensive administration of air pollution.

## CONFLICT OF INTEREST

The authors declare that there are no conflicts of interest.

## AUTHOR CONTRIBUTIONS

HK, YS and YZ conceived the study. WH, HP and HK conducted the experiments. JM assisted in ICP‐MS analysis. JZ assisted in synchrotron‐based XRF microscopy. QW, AL, HK, YS and YZ analysed the data and wrote the paper. All authors have reviewed the final version of the manuscript and approve it for publication.

## Supporting information

Supporting Information

## Data Availability

The data that support the findings of this study are available from the corresponding author upon reasonable request.
