# Supplementary material for: Autophagy changes in lung tissues of mice at 30 days after carbon black‐metal ion co‐exposure
Source: Cell Prolif. 2020 Jun 9;53(7):e12813. doi: 10.1111/cpr.12813 (PMC7377941; doi:10.1111/cpr.12813)
Supplement: Supplementary file 1 — Supporting Information [file CPR-53-e12813-s001.docx]

**Table S1.** The size of CBs, CBs-Ni clusters in different medium determined by DLS. All the particles exhibited a narrow size distribution, with polydispersity indices ranging from 0.1 to 0.3.

|  | Mean particle size (nm) | |
| --- | --- | --- |
|  | CBs | CBs-Ni |
| MilliQ Water | 97 ± 28 | 118 ± 36 |
| Saline (0.05 % tween 80) | 110 ± 33 | 155 ± 48 |


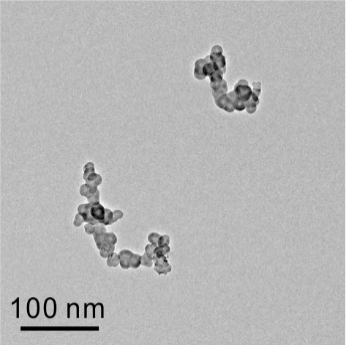


**Figure S1.** TEM images of CBs dispersed in MilliQ water.


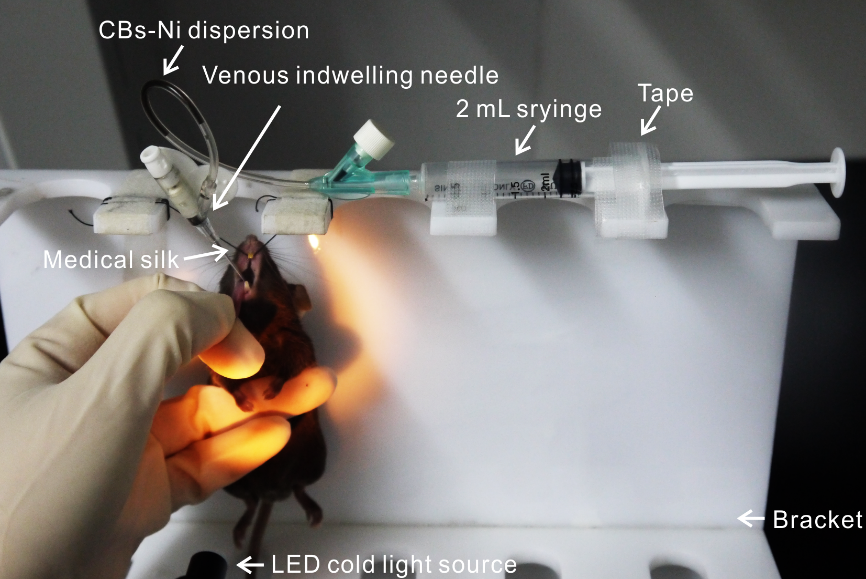


**Figure S2.** Rapid, non-surgical, and efficient intratracheal instillation in C57BL6 mice.


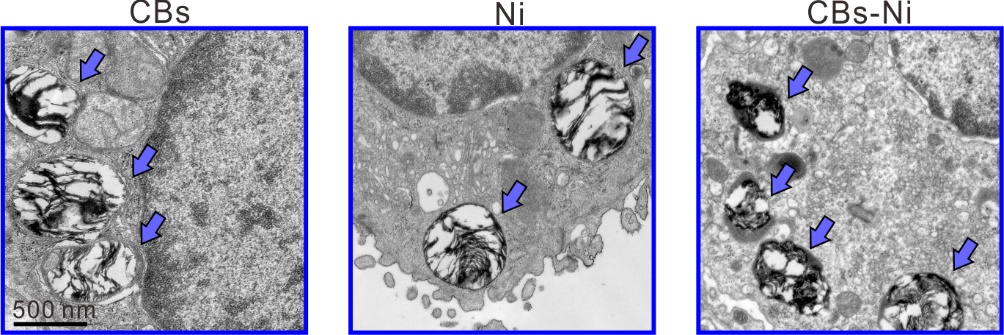


**Figure S3.** TEM images of lung tissue sections of mice at 7 d post-instillation. C57BL6 mice were instilled with CBs,Ni or CBs-Ni mixture. Mitochondria are indicated with blue arrows.


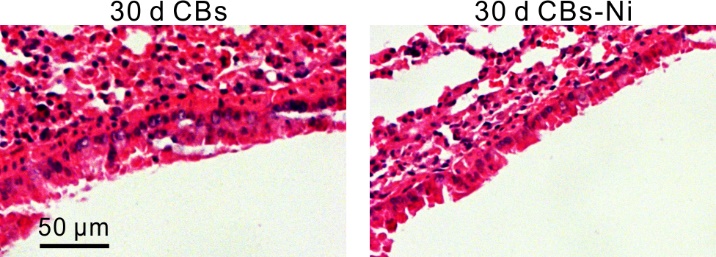


**Figure S4.** Hematoxylin and eosin (H&E) histopathological sections of lung tissues were analyzed at 30 d post-instillation (n = 3). C57BL6 mice were instilled with CBs or CBs-Ni mixture.


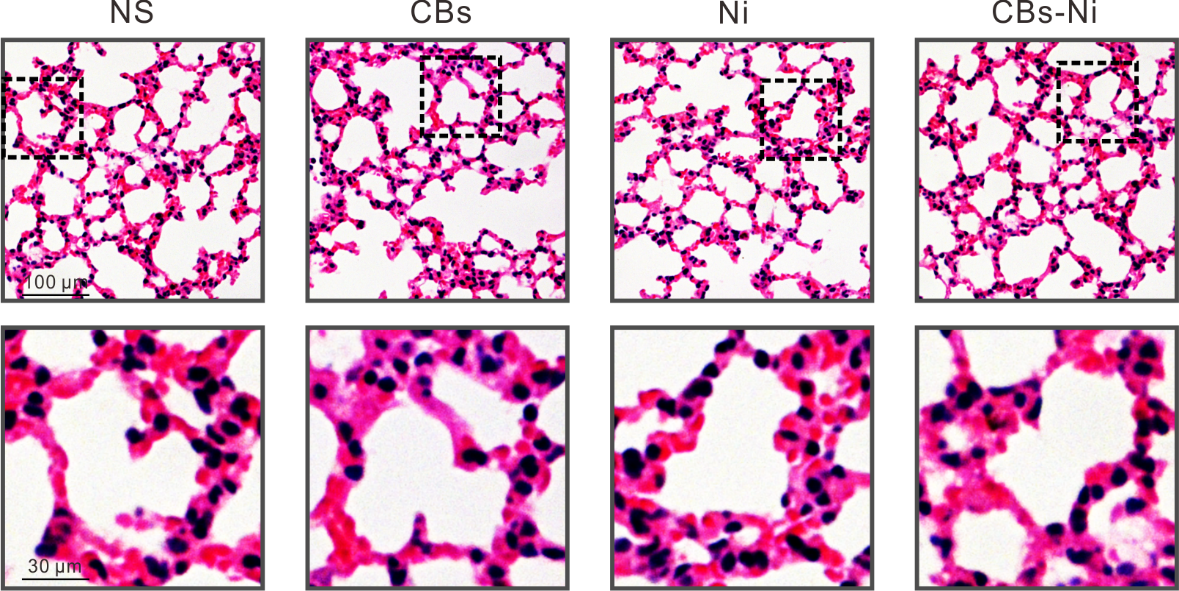


**Figure S5.** Hematoxylin and eosin (H&E) histopathological sections of lung tissues were analyzed at 30 d post-instillation (n = 3). C57BL6 mice were instilled with CBs, Ni or CBs-Ni mixture. The bottom panel is high-magnification images of the indicated portion.


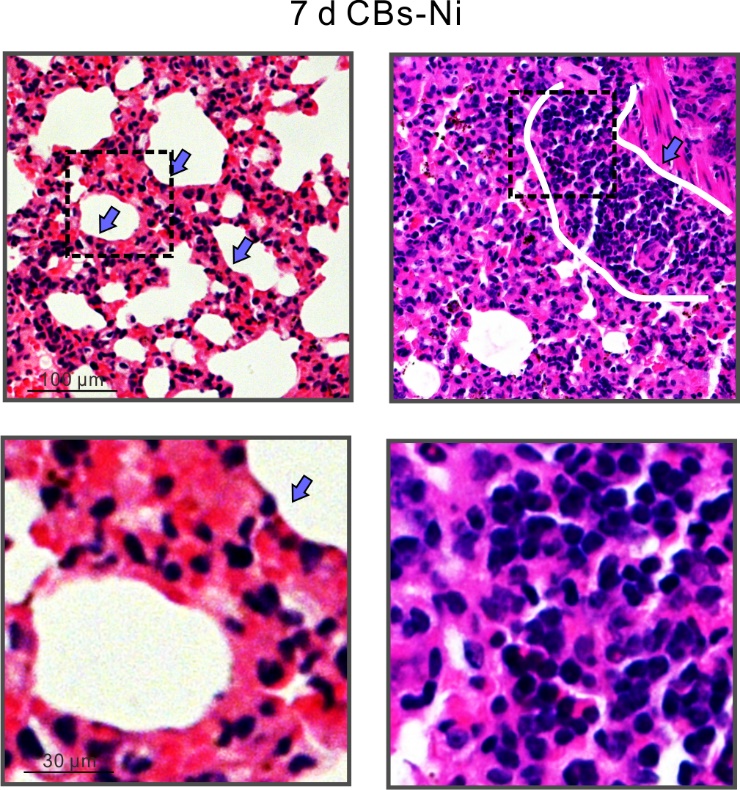


**Figure S6.** Hematoxylin and eosin (H&E) histopathological sections of lung tissues were analyzed at 7 d post-instillation (n = 3). C57BL6 mice were instilled with CBs-Ni mixture. The bottom panel is high-magnification images of the indicated portion.


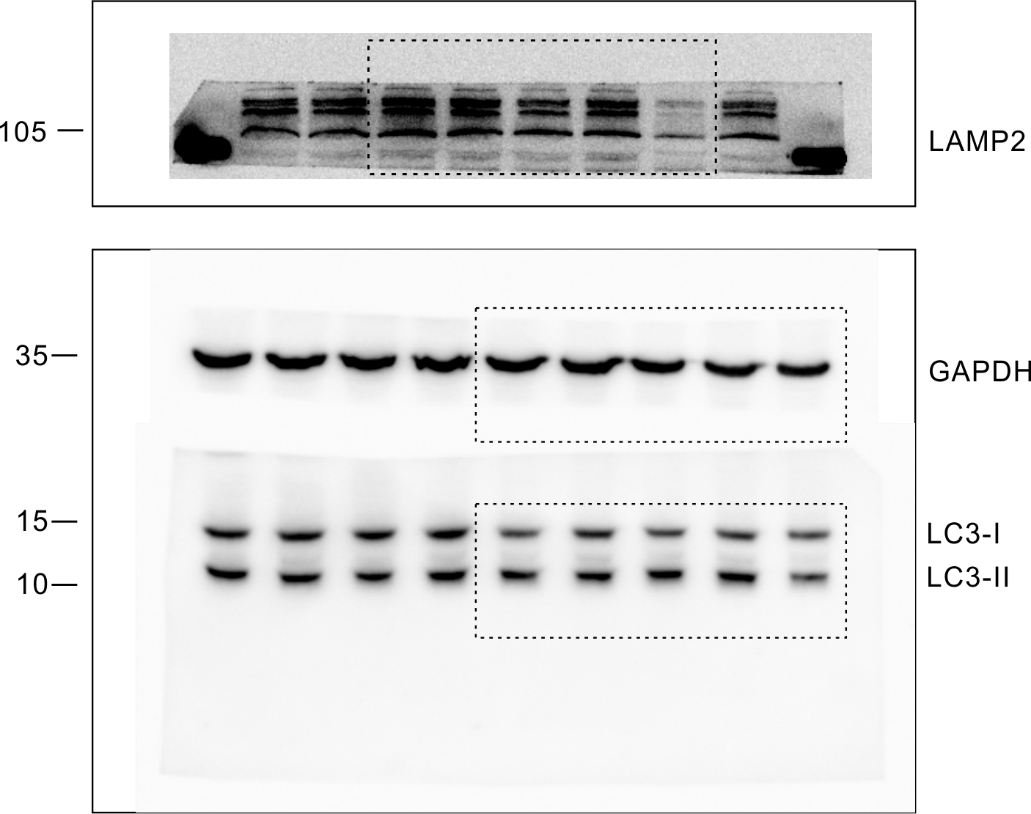


**Figure S7.** Uncropped scans of western blots included in figure 2.
